# Peripheral 5-HT3 Receptors Are Involved in the Antinociceptive Effect of Bunodosine 391

**DOI:** 10.3390/toxins10010012

**Published:** 2017-12-27

**Authors:** Wilson Alves Ferreira Junior, Andre Junqueira Zaharenko, Kohei Kazuma, Gisele Picolo, Vanessa Pacciari Gutierrez, Jose Carlos de Freitas, Katsuhiro Konno, Yara Cury

**Affiliations:** 1 Laboratory of Pain and Signaling, Butantan Institute, Av. Vital Brasil, 1500, 05503-900 São Paulo, SP, Brazil; ferreirajr@usp.br (W.A.F.J.); gisele.picolo@butantan.gov.br (G.P.); vangutierrez@gmail.com (V.P.G.); 2 Laboratory of Genetics, Butantan Institute; Av. Vital Brasil, 1500, 05503-900 São Paulo, SP, Brazil; a.j.zaharenko@gmail.com; 3 Institute of Natural Medicine, University of Toyama, Sugitani 2630, Toyama 930-0194, Japan; cokazuma@inm.u-toyama.ac.jp (K.Ka.); kkgon@inm.u-toyama.ac.jp (K.Ko.); 4 Department of Physiology, Bioscience Institute, University of Sao Paulo, Rua do Matão, trav. 14, 321, 05508-090 São Paulo, SP, Brazil; jfreitas@usp.br

**Keywords:** BDS 391, sea anemone, antinociception, peripheral 5-HT3 receptors, overt pain, neuropathic pain, inflammatory hyperalgesia

## Abstract

Bunodosine 391 (BDS 391), a low molecular weight compound isolated from the sea anemone *Bunodosoma cangicum*, increases the nociceptive threshold and inhibits inflammatory hyperalgesia. Serotonin receptors are involved in those effects. In this study, we have expanded the characterization of the antinociceptive effect of BDS 391 demonstrating that, in rats: (a) the compound inhibits (1.2–12 ng/paw) overt pain, in the formalin test, and mechanical hyperalgesia (0.6–6.0 ng/paw) detected in a model of neuropathic pain; (b) intraplantar administration of ondansetron, a selective 5-HT3 receptor antagonist, blocks the effect of BDS 391, whereas ketanserin, a 5-HT2 receptor antagonist, partially reversed this effect, indicating the involvement of peripheral 5-HT2 and 5-HT3 receptors in BDS 391 antinociception; and (c) in binding assay studies, BDS 391 was not able to displace the selective 5-HT receptor antagonists, suggesting that this compound does not directly bind to these receptors. The effect of biguanide, a selective 5-HT3 receptor agonist, was also evaluated. The agonist inhibited the formalin’s nociceptive response, supporting an antinociceptive role for 5-HT3 receptors. Our study is the first one to show that a non-peptidic low molecular weight compound obtained from a sea anemone is able to induce antinociception and that activation of peripheral 5-HT3 receptors contributes to this effect.

## 1. Introduction

Sea anemone venoms are a rich source of protein and peptide toxins, which have been characterized as pore forming membrane toxins (16–20 kDa) [1,2,3], serine protease inhibitors of Kunitz/BPTI family (6–7 kDa) [4,5,6] or neurotoxins acting on Kv, NaV, ASIC or TRPV channels [7,8,9,10,11,12]. Mainly in the last two decades, experimental studies have demonstrated that, in addition to proteins and peptides, these venoms contain low (smaller than 1 KDa) molecular weight compounds [13]; however, the biological activity of these molecules is still not fully characterized. Zaharenko et al., in 2011, isolated from the venom of the Brazilian sea anemone *Bunodosoma cangicum*, a low molecular weight (391 Da) and non-peptidic compound named Bunodosine 391 (BDS 391), composed of a bromoindole group connected to a histidine [14]. Based on the structural similarity of the 6-bromoindole-3-acetic acid moiety of BDS 391 to serotonin (5-HT) and data from the literature indicating that serotonin is involved in a variety of acute and chronic pain states [15,16], we have previously investigated the possible nociceptive effect of BDS 391. Interestingly, this compound, peripherally administered in low doses to rats, increases, per se, the nociceptive threshold of the animals and also inhibits inflammatory hyperalgesia induced by carrageenan. These effects were not due to activation of opioid receptors or to a possible anti-inflammatory action of BDS 391, but mediated by activation of serotoninergic receptors [14]. 

The role of serotonin in pain and analgesia, although controversial, is well recognized. The effect of 5-HT on nociception depends on the subtype of receptor activated by this amine and also on the localization (central or peripheral) of the receptors in the nervous system [15,16,17,18]. The 5-HT receptors belong to a family of seven receptors (5-HT1 to 5-HT7) that are subdivided into 14 subtypes [19]. These receptors, with the exception of the 5-HT3 receptors, are G-protein coupled receptors. 

Serotonin—peripherally administered to rats and humans, or released as an endogenous mediator during inflammation or neuronal injury—induces pain by activation of, mainly, 5-HT1, 5-HT2 and 5-HT3 receptors [20,21,22,23,24]. Recent data have also indicated that 5-HT4, 5-HT6 and 5-HT7 receptors participate in allodynia and hyperalgesia detected in inflammatory and in chronic pain states [25,26,27,28]. Despite these data, it is important to stress that several lines of evidence have indicated that 5-HT amplifies the nociceptive effects of algesic mediators rather than causing nociception per se [15,29,30]. In contrast to those data, Tzeng et al., in 2017 [31], demonstrated, in rats, that subcutaneous injection of serotonin causes, in a dose-dependent manner, a local inhibition of the cutaneous trunci muscle reflex induced by skin pinprick, indicating an antinociceptive effect for this amine. In this sense, Diniz et al., in 2015 [18], have also demonstrated, in mice, that peripheral (intraplantar) administration of low doses of serotonin inhibits prostaglandin E2-induced hyperalgesia by activation of 5-HT1B, 5-HT2A and 5-HT3 receptors. In addition to the data from Diniz et al., the role of 5-HT1B/1D receptors in analgesia was also demonstrated in models of thermal hyperalgesia induced by carrageenan [32]. Altogether, these findings support the involvement of serotonin and its receptors on nociceptive pathways in the periphery. However, pain enhancing or antinociceptive actions of 5HT rely on the type of receptor involved, the presence of peripheral sensitization and the site of serotonin’s action.

In the central nervous system, activation of 5-HT receptors inhibits pain, through activation of the descending inhibitory system [33]. Peng et al. (1996) [34] and Kiefel et al. (1992) [35], showed that serotonin, released in low doses by stimulation of the periaqueductal gray, or by the action of morphine in this site, is involved in antinociception, by activation of 5-HT2 and 5-HT3 receptors in the dorsal horn of the spinal cord. Besides those receptors, activation of 5-HT1A receptors in the spinal cord also inhibits nociception [36]. Recently, an important antinociceptive role for the central 5-HT7 receptors in neuropathic pain in mice, by a mechanism dependent on the anterior cingulate cortex, was also demonstrated [37]. Despite this well demonstrated central antinociceptive effect, nociceptive phenomena after activation of 5-HT3 receptors in the CNS have also been observed [38]. In contrast to the well established antinociceptive effect of 5-HT in the Central Nervous System, some consistent experimental data have indicated a nociceptive role for serotonin and its contribution to the pain descending facilitatory pathway, through activation of 5-HT1 and 5-HT3 receptors, mainly in the spinal cord [39,40].

Taken together, these data demonstrate the complexity of action of serotonin and its receptors on pain systems. The two opposite effects—nociception/antinociception—of serotonin are mainly a result of activation of different 5-HT receptor subtypes, the concentration and site of serotonin release and the type of nociceptive phenomena.

Based on our initial data on the involvement of serotonin receptors in the antinociceptive effect of BDS 391 and on the controversial data on the role of serotonin receptors in pain and analgesia, this work was undertaken to further characterize the involvement of 5-HT receptors in the peripheral antinociceptive effect of BDS 391. Our results indicate that peripheral 5-HT3 receptors are involved in the effect of BDS391, in different behavioral models of pain. Furthermore, the potential antinociceptive action of 5-HT3 receptor activation, in the periphery, is supported by the use of a selective agonist of these receptors in these same models of pain evaluation.

## 2. Results

### 2.1. Characterization of the Antinociceptive Effect of BDS 391

We have previously demonstrated that BDS 391, administered by intraplantar (i.pl.) route to naive rats, increases the nociceptive threshold of the animals to mechanical stimulation [14]. To confirm that BDS 391 features antinociceptive activity, we have determined, in the present study, the possible effect of the compound in different models of acute and chronic pain. 

**Figure 1:**
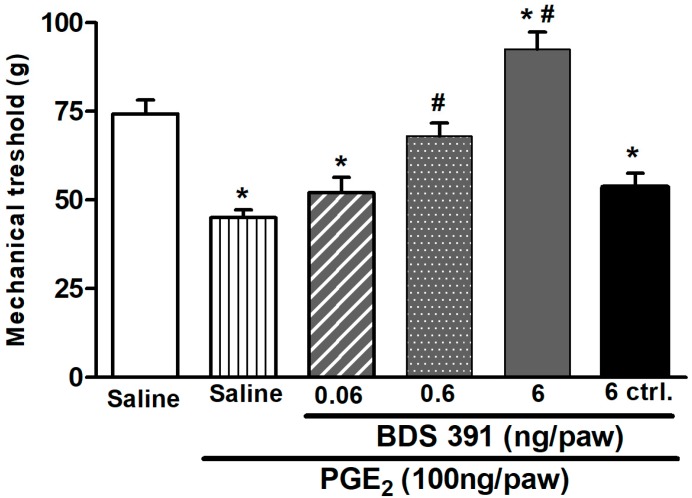
Dose–response curve of the inhibitory effect of BDS 391 on hyperalgesia induced by PGE_2_. Nociceptive threshold was recorded in the rat paw pressure test applied before and 3 h after administration of PGE_2_ (100 ng/paw) or saline (control). BDS 391 or saline (control) was injected subcutaneously (i.pl.) 150 min after PGE_2_. BDS 391 (0.006–6 ng/paw) administered in the ipsilateral paw, inhibited, in a dose-dependent manner, PGE_2_-induced hyperalgesia, while saline has no effect. Injection of BDS 391 (6 ng/paw) into the contralateral (ctrl) paw did not alter PGE_2_-induced hyperalgesia. Data represent mean values ± S.E.M. for five rats per group. * Values significantly different from those before PGE2 injection and ^#^ values significantly different from those of the control group (PGE_2_ + saline) (*p* < 0.05).

The effect of BDS 391 was first assayed in the model of prostaglandin E_2_ (PGE_2_)-induced hyperalgesia. The intraplantar injection of prostaglandin E_2_ caused a significant decrease in the nociceptive threshold, detected 3 h after treatment, as compared to basal values, thus characterizing the phenomenon of hyperalgesia. BDS 391 (0.06–6 ng/paw), injected 150 min after PGE_2_, caused a dose–response inhibition of PGE_2_-induced hyperalgesia, as well as an increase in the mechanical threshold to values above those recorded at baseline (Figure 1). Administration of BDS 391 (6 ng/paw) into the contralateral paw of the animals did not interfere with the ipsilateral PGE_2_-induced hyperalgesia, indicating that, at the doses presently used, BDS 391 induces only a local (peripheral) antinociceptive effect.

**Figure 2:**
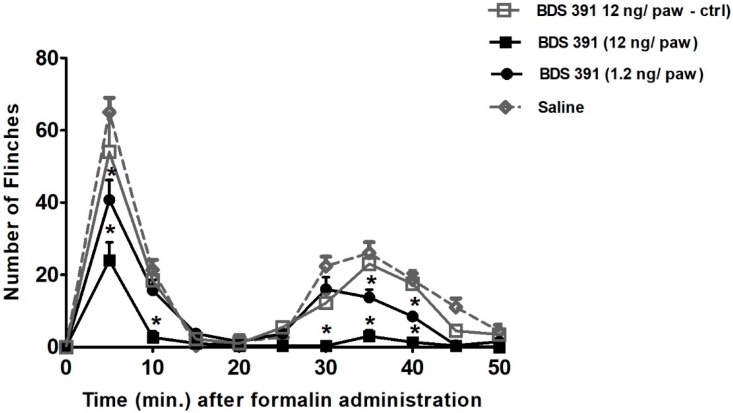
Dose–response curve of the inhibitory effect of BDS 391 on the nociceptive response induced by formalin administration into mice hind paw, evaluated as the number of flinches. Formalin (1%) subcutaneously administered into the mice right hind paw produced repeated flinching behavior characterized by a biphasic time course. Immediately after formalin administration, phase 1 of the nociceptive response began. Gradual diminishing of this response lasted approximately 10 min. Twenty minutes after formalin administration, phase 2 began and went on for about 50 min. BDS 391 (1.2 and 12 ng/paw) administered 30 min before formalin, inhibited, in a dose-dependent manner, both phases of formalin nociception. Intraplantar administration of saline (control) did not interfere with the nociceptive response induced by formalin. Data are expressed as the mean ± S.E.M. of at least six animals per group. * Significant difference (*p* < 0.05, ANOVA) from saline group (control).

The effect of the compound was also evaluated in the formalin test, an experimental model of overt pain. Formalin (1%) subcutaneously administered into the mice right hind paw produced repeated flinching behavior characterized by a biphasic time course (Figure 2). Immediately after formalin administration, phase 1 of the nociceptive response began. Gradual diminishing of this response lasted approximately 10 min. Twenty minutes after formalin administration, phase 2 began and went on for about 50 min. 

Ipsilateral injection of BDS 391 (1.2–12 ng/paw), administered 30 min before formalin, significantly inhibited, in a dose-dependent manner, both phases of formalin-induced flinching behavior (Figure 2). The BDS 391 at the highest dose could completely inhibit formalin-induced phase 2 nociception. Contralateral injection of BDS 391 (12 ng/paw) did not interfere with nociception induced by the ipsilateral injection of formalin, indicating that, at the doses presently used, BDS 391 induces only a local (peripheral) antinociceptive effect.

**Figure 3:**
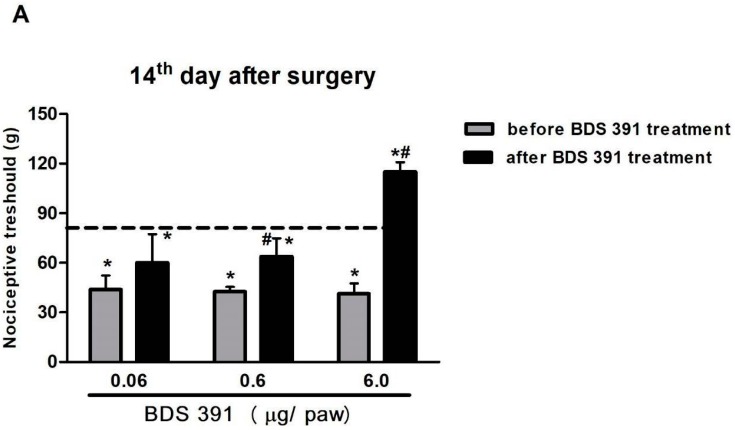

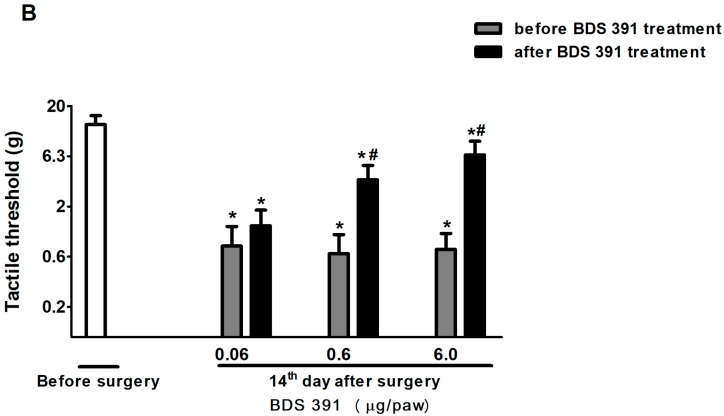
Dose–response curve of the inhibitory effect of BDS 391 on hyperalgesia and allodynia induced by rat sciatic nerve chronic constriction. For induction of nerve injury, four ligatures were loosely tied around the exposed common sciatic nerve. Hyperalgesia was determined using the rat paw pressure test (**A**); and low-threshold mechanical allodynia was measured using the von Frey hair filaments (**B**). Tests were applied before and 14 days after nerve ligation. On Day 14, the tests were applied before and 30 min after i.pl. injection of BDS 391 (0.06, 0.6 or 6 µg/paw) or saline (control group). Data represent mean values ± S.E.M. for four rats. ^#^ Values significantly different from those of control (without BDS 391 treatment) group; * Values significantly different from those obtained before surgery (*p* < 0.05).

We also investigated the effect of BDS 391 in a model of chronic pain induced by chronic constriction of rat sciatic nerve. The constriction injury induced a marked decrease in the mechanical threshold (Figure 3A), and also lowered withdrawal threshold responses to the von Frey hairs (Figure 3B). One day after nerve ligation, mechanical hyperalgesia and low-threshold mechanical allodynia were detected. Both phenomena lasted for a minimum of 14 days. The intact contralateral paw did not show changes in pain threshold (data not shown). 

Hyperalgesia (Figure 3A) induced by nerve constriction was inhibited, in a dose-dependent manner, by BDS 391 administered i.pl. on Day 14 after surgery, 30 min before nociceptive evaluation. The compound, at the higher dose (6 µg/paw), also increases mechanical threshold to values above those recorded at baseline (i.e., antinociception) (Figure 3A). BDS 391 partially, but significantly, inhibited, in a dose-dependent manner, allodynia induced by nerve injury (Figure 3B).

### 2.2. Serotonin Receptors Are Involved in the Antinociceptive Effect of BDS 391

We have previously demonstrated that the rise in the basal nociceptive threshold of the rats caused by BDS 391 was prevented by methysergide, a non-selective serotonin receptor antagonist [14]. To confirm and further investigate the role of these receptors in the antinociceptive effect of BDS 391, selective antagonists of 5-HT1, 5-HT2 and 5-HT3 serotonin receptors were intraplantarly administered, 15 min before BDS 391 (6 ng/paw) treatment.

**Figure 4:**
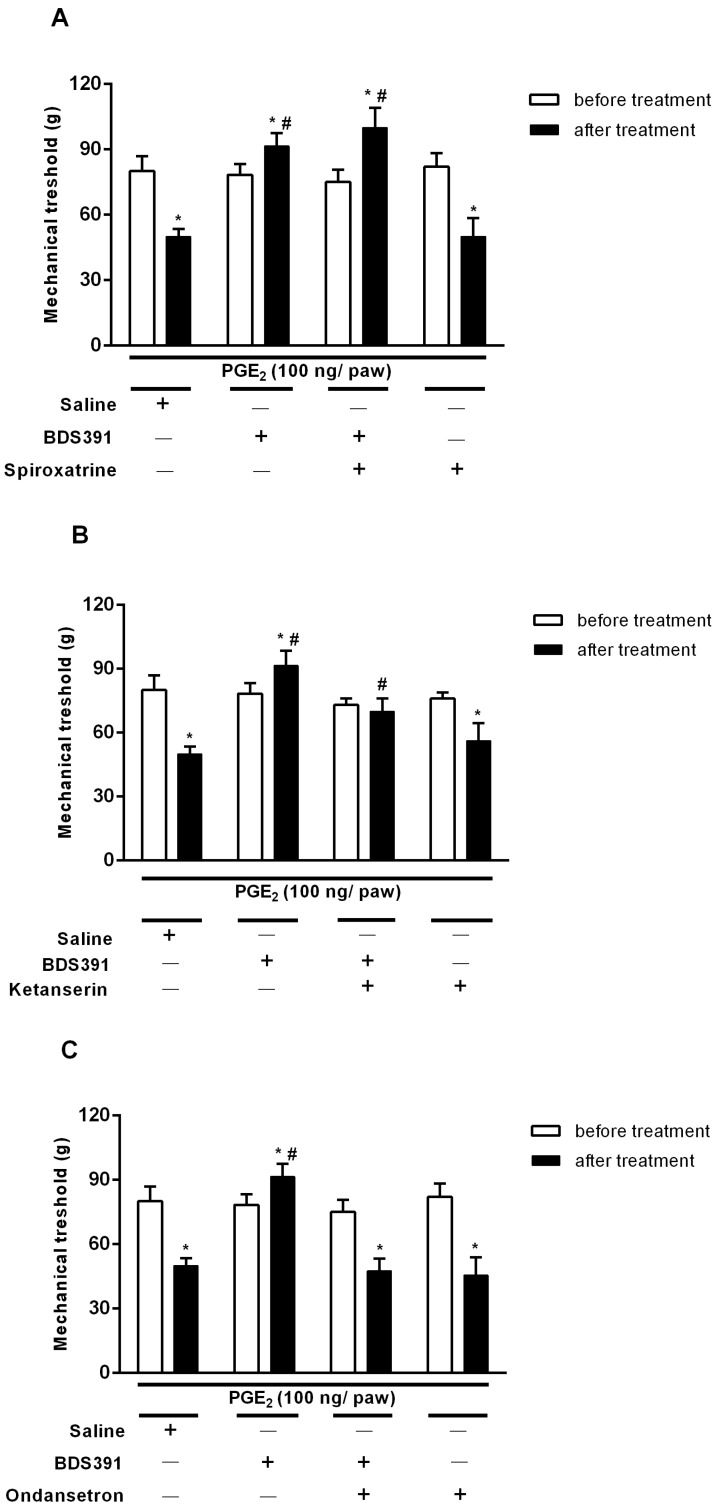
Involvement of peripheral serotoninergic receptors in the antinociceptive effect of BDS 391 (6 ng/paw), in the model of PGE_2_-induced hyperalgesia. BDS 391 was administered by i.pl. route, 150 min after the PGE_2_ (100 ng/paw). The animals were treated with: (**A**) Spiroxatrine (250 µg/paw), a 5-HT1A selective receptor antagonist; (**B**) Ketanserin (250 μg/paw), a selective 5-HT2 receptor antagonist; and (**C**) Ondansetron (250 µg/paw), a 5-HT3 selective receptor antagonist. Serotonin antagonists were administered by i.pl. route 15 min before BDS 391. Saline was used as control. Mechanical threshold was measured before and 3 h after PGE_2_ injection. * *p* < 0.05 as compared to baseline values. ^#^ *p* < 0.05 as compared to the control group (Saline).

In the PGE_2_-induced hyperalgesia, the antinociceptive activity of BDS 391 was abolished by ondansetron, a selective antagonist of 5-HT3 receptors, while ketanserin, a 5-HT2 receptor antagonist, partially reversed the BDS 391 effect. On the other hand, spiroxatrine, an antagonist of 5-HT1 serotonin receptors, did not alter the action of the compound (Figure 4). Based on the results obtained with ondansetron, and to further confirm the involvement of 5-HT3 receptors, we carried out assays using MDL72222, a selective 5-HT3 receptor antagonist. MDL72222 also inhibits the antinociceptive effect of BDS 391 (PGE_2_-injected rats: 47 g ± 1.2; BDS 391+ PGE_2_-injected rats: 77 g ± 3.6; MDL72222 + BDS 391 + PGE_2_-injected rats: 49 g ± 1.8), confirming the involvement of 5-HT3 receptors in the peripheral antinociceptive action of the compound.

Based on these results demonstrating the involvement of 5-HT3 receptors on the local antinociceptive effect of BDS 391, we further characterized the role of these receptors on the effect of the compound in the neuropathic pain model and formalin test.

**Figure 5:**
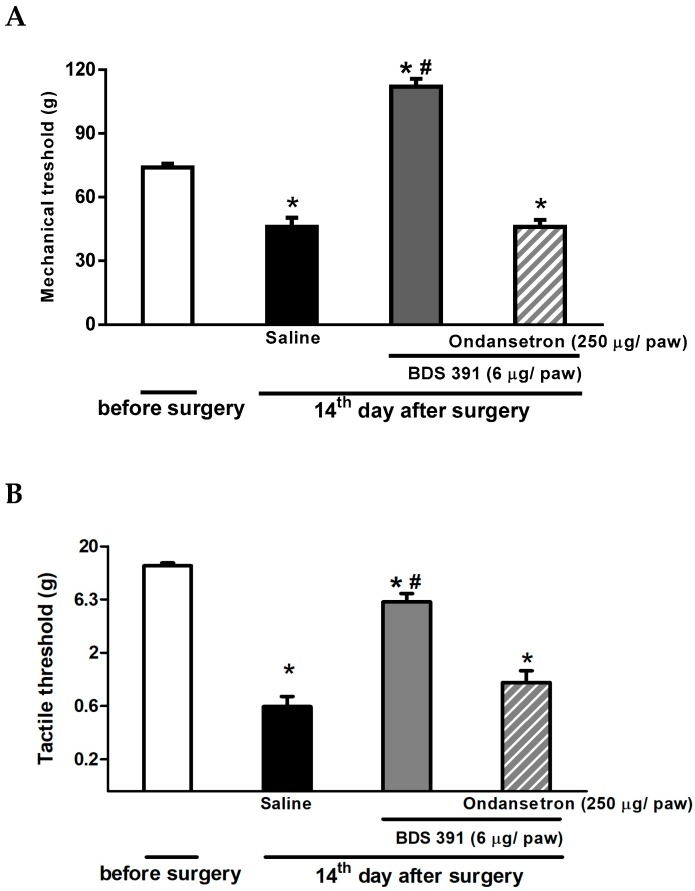
Peripheral 5-HT3 receptors contribute to BDS 391-induced antinociception on chronic constriction injury of the rat sciatic nerve. For induction of nerve injury, four ligatures were loosely tied around the exposed common sciatic nerve. Hyperalgesia was determined using the rat paw pressure test (**A**); and low-threshold mechanical allodynia was measured using the von Frey hair filaments (**B**). Tests were applied before and 14 days after nerve ligation. On Day 14, the tests were applied before and 30 min after i.pl. injection of BDS 391 (6 µg/paw). Ondansetron (250 µg/paw) or saline (100 µL/paw) was intraplantarly injected 15 min before BDS 391. Data represent mean values ± S.E.M. for five rats. ^#^ Values significantly different from those of control group; * Values significantly different from those obtained before surgery (*p* < 0.05).

**Figure 6:**
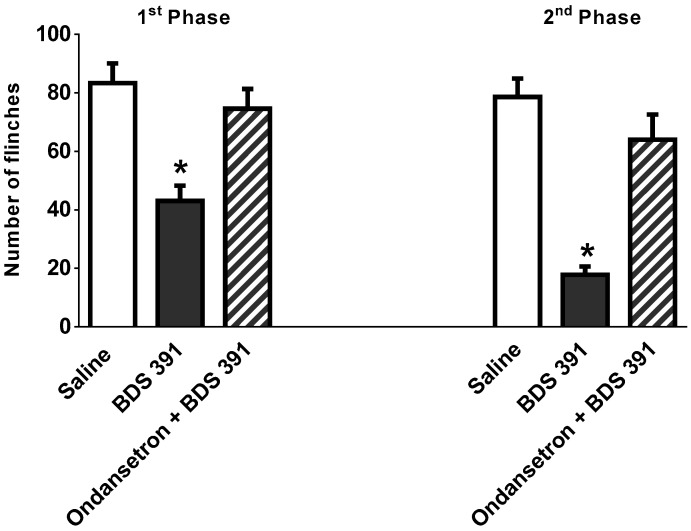
Peripheral 5-HT3 receptors contribute to the antinociceptive effect of BDS 391 on formalin test. Nociceptive responses induced by formalin (1%) administration into mice hind paw was evaluated as the number of flinches. Subcutaneous formalin (1%) injection induced a pattern of flinching behavior characterized by a biphasic time course (Figure 2). Phase 1 of the nociceptive response began immediately after formalin administration and then diminished gradually during approximately 10 min. Phase 2 began around 20 min after formalin administration and lasted about 50 min. BDS 391 (12 ng/paw) was administered 30 min before formalin injection. Ondansetron (250 µg/paw) or saline (20 µL/paw) was injected by intraplantar route 15 min before BDS 391. Data are expressed as the mean ± S.E.M. of at least six animals. * Significant difference (*p* < 0.05, ANOVA) from saline group (control).

Intraplantar administration of ondansetron abolished the antihyperalgesic and antiallodynic effects of BDS 391 in the model of chronic constriction injury of the sciatic nerve (Figure 5A,B). The antagonist also inhibited the antinociceptive effect of BDS 391 in the formalin test (Figure 6).

The antagonists per se, in the doses presently used, did not interfere with nociception caused by PGE_2_, formalin or chronic constriction of the sciatic nerve (Figure 4, Figure 5 and Figure 6).

Taken together, these results indicate the involvement of peripheral 5-HT3 receptors in the antinociceptive effect of BDS 391. 

### 2.3. Antinociceptive Effect of Biguanide, a 5-HT3 Receptor Agonist

**Figure 7:**
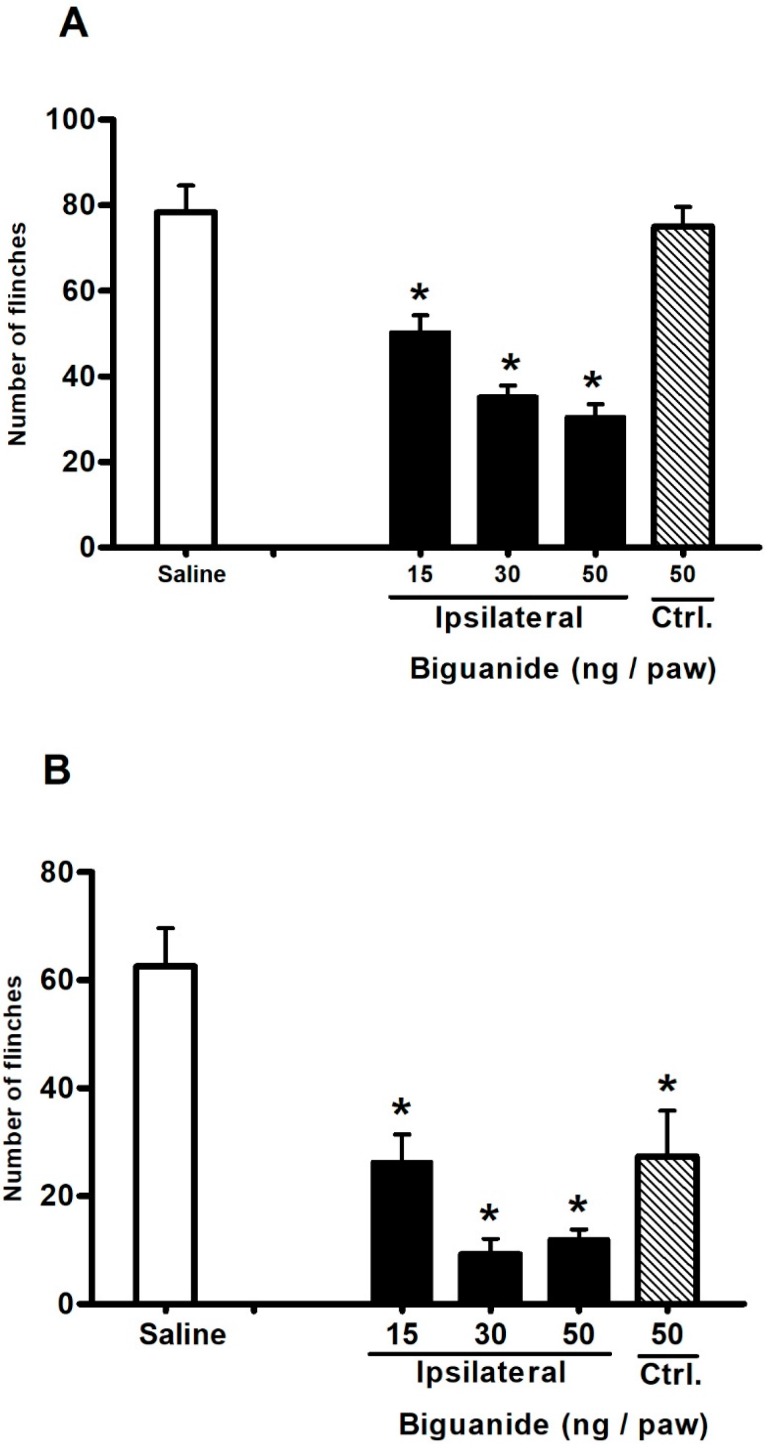
Effect of Biguanide, a selective 5-HT3 receptor agonist, on the nociceptive response induced by formalin (1%) administration into mice hind paw. Nociception was evaluated as the number of flinches. Formalin (1%) subcutaneously administered into the mice right hind paw produced repeated flinching behavior characterized by a biphasic time course. Immediately after formalin administration, phase 1 of the nociceptive response began (**A**). Gradual diminishing of this response lasted approximately 10 min. Twenty minutes after formalin administration, phase 2 began and went on for about 50 min (**B**). Biguanide administered 30 min before formalin inhibited, in a dose-dependent manner, both phases of formalin nociception. Injection of the agonist (50 ng/paw) into the contralateral (ctrl) paw interferes with the second phase of the nociceptive response induced by formalin. Intraplantar administration of saline (control) did not interfere with the nociceptive response induced by formalin. Data are expressed as the mean ± S.E.M. of at least six animals per group. * Significant difference (*p* < 0.05, ANOVA) from saline group (control).

Based on the results showing that peripheral 5-HT3 receptors are involved in the antinociceptive effect of BDS 391, we have herein evaluated whether peripheral activation of these receptors are able to induce antinociception. For this purpose, we investigated the effect of Biguanide, a selective agonist of 5-HT3 receptors, administered by intraplantar route, on nociception caused by formalin. The results indicate that this agonist (15–50 ng/paw) partially inhibited, in a dose-dependent manner, formalin-induced nociception, interfering with both phases of the response (Figure 7A,B). The agonist in the highest dose was administered in the contralateral paw. The results showed that the agonist interferes with the second, but not the first, phase of the nociceptive response, indicating that, at the highest dose, the agonist induces a systemic effect.

### 2.4. BDS 391 Did Not Directly Bind to 5-HT3 Receptors

Based on the results demonstrating the involvement of 5-HT3 receptors in the antinociceptive effect of BDS 391, we evaluated the possible direct binding of the compound to serotonin receptors.

**Figure 8:**
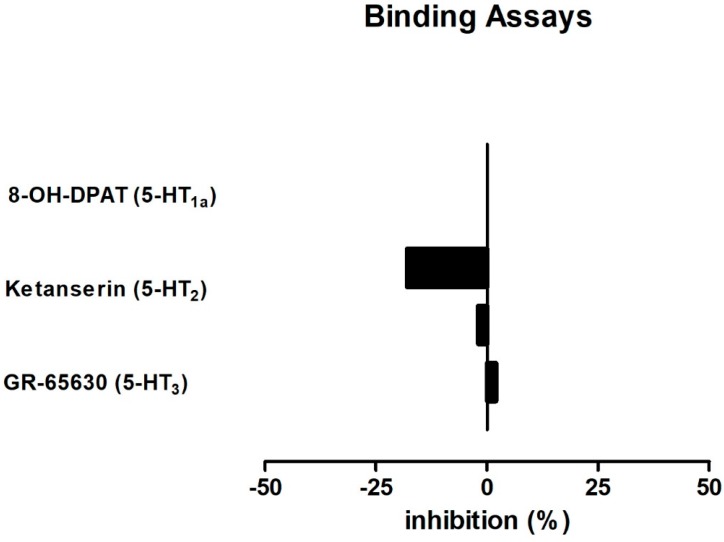
Binding affinity studies were carried out by Ricerca Biosciences LCC, (Pharmacology Laboratories 158 Li-The Road, Peitou Taipei, Taiwan 112. Taiwan R.O.C.) using its standard protocols. In this study, specific binding of BDS 391 to CHO-K1 or HEK-293 cell membranes expressing the 5-HT1, 5-HT2 and 5-HT3 serotonin receptor subtypes was determined. Human recombinant CHO-K1 and HEK 293 cells membranes were incubated for 1 h at 25 °C, with 50 mM Tris-HCl, pH 7.4, 1 mM EDTA, 5 mM MgCl buffer containing the radioligands [^3^H] 8-OH-DPAT (1.5 nM), [^3^H] Ketanserin (0.5 nM) or [^3^H] GR-65630 (0.69 nM), antagonists of, respectively, 5-HT1, 5-HT2 and 5-HT3 serotonin receptors. Non-specific binding was determined using 10 µM Metergoline, 1 µM Mianserin or 10 µM MDL 72222, respectively. For the dissociation assays, BDS 391 was added to the incubation medium in concentrations up to 2 µM. Radioactivity was determined by scintillation counting. Responses were considered significant for 50% inhibition or stimulation. In our experimental conditions, no significant responses were detected.

The results obtained in the binding assays showed that BDS 391 (2 μM) was not able to displace the binding of the selective 5-HT3 receptors radioligand, [^3^H] GR65630 (0.69 nM). In this assay, we have also evaluated the possible binding of the compound to 5-HT1 and 5-HT2 receptors. The results also demonstrated that BDS 391 is not able to displace the binding of [^3^H] 8-OH-DPAT (1.5 nM) and [^3^H] Kentanserin (0.5 nM), respectively, to 5-HT1A and 5-HT2 selective receptor ligands (Figure 8).

## 3. Discussion

We have previously demonstrated that BDS 391, a low molecular weight compound first isolated from the Brazilian sea anemone *Bunodosoma cangicum*, peripherally administered in low doses to rats, increases, per se, the nociceptive threshold of the animals and inhibited inflammatory hyperalgesia induced by carrageenan. This effect was not due to activation of opioid receptors or to a possible anti-inflammatory action of the compound, but mediated by activation of serotonin receptors [14]. In this study, we have expanded the characterization of the antinociceptive effect of BDS 391, demonstrating the following: (a) In addition to the potent antinociceptive effect in acute inflammatory hyperalgesia, BDS 391 significantly inhibits (1.2–12 ng/paw) overt pain, evaluated in the formalin test, and mechanical hyperalgesia (0.6–6.0 ng/paw) detected in a model of chronic (neuropathic) pain. (b) Peripheral 5-HT2 and 5-HT3 receptors are involved in the antinociceptive effect of BDS 391, in a model of PGE_2_-induced hyperalgesia. This suggestion is based on the results showing that intraplantar administration of ondansetron, a selective antagonist of 5-HT3 receptors, blocks the antinociceptive effect of BDS 391, whereas ketanserin, a 5-HT2 receptor antagonist, partially reversed this effect. The contribution of 5-HT3 receptors for the antinociceptive effect of BDS 391 was further confirmed in the models of overt (formalin test) and neuropathic pain. (c) The involvement of peripheral serotoninergic receptors in the antinociceptive effect of BDS391 is not due to a direct binding of this compound to these receptors, because binding assays did not show displacement of the labeled receptor antagonist by BDS 391. In addition, despite the controversial data on the effect of 5-HT3 receptors on the nociceptive system, our data support an antinociceptive role for 5-HT3 receptors, as intraplantar administration of Biguanide, a selective 5-HT3 receptor agonist, significantly inhibited the nociceptive response (both phases) of formalin.

In this study, we showed a peripheral (local) antinociceptive action of BDS 391 in the prostaglandin E_2_-induced hyperalgesia. These data indicate a direct action of the compound on the activity of the afferent sensory nerve, because PGE_2_ directly sensitizes the primary sensory neuron by interacting with receptors coupled to second messengers, decreasing the pain threshold of the animals [41,42,43]. We have also shown that the antinociceptive effect of BDS 391 in this model of hyperalgesia is mediated by activation of peripheral 5-HT receptors. The involvement of 5-HT receptors in the nociceptive system is well demonstrated in the literature, being the type of effect—nociception or antinociception—dependent on the type and localization of the 5-HT receptor as well as the dose of the serotonin receptor agonist [15]. Here we demonstrated the involvement of the peripheral 5-HT2 (partially) and 5-HT3 receptors in the antinociceptive effect of BDS 391 in the PGE_2_-induced hyperalgesia. Our results are supported by data from the literature demonstrating that inhibition of prostaglandin E_2_-induced hyperalgesia by the peripheral administration of low doses of serotonin is mediated by activation of 5-HT2A and 5-HT3 receptors [18]. It is important to stress that mRNA for serotonin receptors, including 5-HT2 and 5-HT3 receptors, has been detected in dorsal root ganglia, as well as the presence of those receptors in unmyelinated nerve fibers [44,45]. Furthermore, the 5-HT2 and 5-HT3 receptor subtypes are co-expressed in the peripheral nociceptive fibers [46] and interactions between these receptor subtypes have been observed. In this sense, Hu and collaborators [47] have shown that activation of the 5-HT3 receptors can be potentiated by the activation of the 5-HT2 receptor. Therefore, in our experimental conditions, the possible interaction between 5-HT3 and 5-HT2 receptors could be considered.

In addition to the effect on PGE_2_-induced hyperalgesia, our work also suggests that a single dose of BDS 391 inhibits chronic pain, more specifically neuropathic pain caused by chronic constriction injury (CCI) of the rat sciatic nerve. The damage to peripheral nerves in humans often results in chronic neuropathic pain characterized by spontaneous burning pain accompanied by allodynia and hyperalgesia [48]. Our data showed that intraplantar administration of a single dose of BDS 391 inhibits hyperalgesia caused by nerve injury, confirming a local antinociceptive effect for the compound also mediated by activation of peripheral 5-HT3 receptors. Despite the well-characterized role of peripheral and spinal serotonin and 5-HT3 receptors for neuropathic pain genesis [15,49], our data support an antinociceptive activity for the 5-HT3 receptors in the periphery, for this type of pain, as the local administration of the selective antagonist of these receptors blocked the antinociceptive effect of BDS 391. 

We also evaluated the effect of BDS 391 in the formalin test, a model of overt pain widely used for characterization of drugs endowed with antinociceptive activity [50]. The nociceptive response of formalin occurs in two distinct phases: the initial phase (phase 1), characterized as neurogenic nociception, is attributed to the direct effect of formalin on nociceptors [51,52]. After a brief period, the inflammatory phase (phase 2) occurs, being related to the local release of endogenous mediators responsible for the sensitization of the sensory neurons [53,54]. Similar to that observed in the PGE_2_-induced hyperalgesia and neuropathic pain models, BDS 391 interfered with formalin-induced nociception. The compound partially reversed the neurogenic phase (phase 1) of the formalin test and, at the highest dose, it inhibited phase 2 (inflammatory) response. These data support an antinociceptive role for BDS 391, as well as its ability to directly interfere with neuronal excitability. The antinociceptive action of BDS 391 in this model of overt pain is mediated by activation of peripheral 5-HT3 receptors.

Several data have indicated that peripheral serotonin receptors, including 5-HT3 receptors, are involved in the inflammatory phase of the nociceptive response induced by formalin [55,56,57]. The involvement of 5-HT receptors in nociception induced by formalin has been shown based on the use of selective 5-HT receptor subtypes antagonists, indicating that the flogistic agent releases endogenous serotonin. In fact, Nakajima et al. (2009) [57] demonstrated, using microdialysis technique, that 5-HT is released in the site of formalin injection (rat hind paw) in a formalin concentration-dependent manner. Differences in the experimental assays could contribute to the distinct results obtained in our study. It is important to point out that, in our experimental conditions, we did not evaluate the effect of endogenous serotonin, but analyzed the effect of exogenous administration of a compound that is able to induce antinociception through activation of 5-HT3 receptors. Furthermore, experimental studies have shown that the involvement of serotonin receptors in pain and analgesia depends on the 5-HT receptor subtypes involved, their central or peripheral localization, and also on possible functional changes in the receptors in the presence of tissue injury or pharmacological treatments with serotonin modulators [17]. 

Based on the controversial literature data on the nociceptive/antinociceptive effect of 5-HT3 serotonin receptor agonists, and on our results showing that peripheral 5-HT3 receptors are involved in the antinociceptive effect of BDS 391, we carried out another set of experiments aimed at confirming that activation of these receptors induces antinociception. In these experiments, the action of Biguanide, a selective 5-HT3 receptor agonist, was investigated in the formalin-induced pain model. The results showing that the agonist inhibits both phases of the nociceptive response induced by formalin support data on the antinociceptive activity for activated 5-HT3 receptors. However, in our experimental conditions, inhibition of formalin-phase 2 nociceptive response by the highest dose of the agonist could be due to a systemic effect, as agonist administration into the non-inflamed contralateral paw inhibited nociception in the formalin-injected (ipsilateral) paw. 

As pointed out in the Introduction, the 6-bromoindole-3-acetic acid moiety of BDS 391 has structural similarity to serotonin [14]. Furthermore, comparative analysis with classic agonists and antagonists of 5-HT3 receptors [58,59] emphasizes the high similarity between BDS 391 and these compounds, reinforcing the idea of a possible interaction of BDS 391 with the 5-HT3 receptor subtype. The 5-HT3 receptor subtype, in contrast to the other 5-HT receptor subtypes, is a nonspecific ion channel receptor belonging to the nicotine/gamma-aminobutyric (GABA) receptor family. It is an oligomeric complex consisting of five monomers forming a centrally permeable cylindrical body. Five 5-HT3 receptor subunits (5-HT3A–5-HT3E) have been identified [58,60], for review). Despite our experimental evidence on the involvement of 5-HT3 receptors in the antinociceptive effect of BDS 391, a direct interaction of the compound with these receptors could not be confirmed in our study, because the compound was not able to displace, in radioligand binding assays, the binding of labelled selective 5-HT3 receptor antagonist. Nevertheless, these results do not exclude a possible interaction of BDS 391 with another binding site of the 5-HT3 receptor, distinct from the binding site of the selective antagonist of this receptor. Furthermore, even considering that BDS 391 is a quite stable compound and should not be decomposed to release 6-bromo-3-acetic acid under the experimental conditions that were used in this study, a direct binding of this moiety to 5-HT3 receptors could be considered. Future studies to determine structure-activity relationships of the compound will contribute to the characterization of the compound pharmacophore.

It is important to point out that the antagonists used in the behavioral assays, MDL 72222 and, particularly, ondansetron, are considered potent and selective antagonists of neuronal 5-HT3 receptors. These antagonists are able to induce, in a competitive manner, a complete blockade of the peripheral and central 5-HT3 receptors ([60,61,62,63,64], for review). Furthermore, ondansetron has been widely used for the study and functional characterization of these receptors. However, several lines of evidence have indicated that the 5-HT3 receptor antagonists can act on the other receptors of the Cys-loop receptor family of ligand-gated ion channels, mainly the nicotinic receptors [65,66,67]. Despite these data, experimental evidence has shown that: (a) the 6-bromoindole-3-acetic acid moiety of BDS 391 displays structural similarity to serotonin [14]; (b) in the present study, both ondansetron and MDL 72222 inhibit the antinociceptive effect of BDS 391, whereas biguanide, a selective 5-HT3 receptor agonist [68,69], exhibits peripheral antinociceptive effect; (c) peripheral administration of ketanserin partially inhibits the effect of BDS 391, indicating that 5-HT2 receptors also contribute to the effect of BDS-391; and (d) 5-HT3 and 5-HT2 receptors can coexist and activation of 5-HT2 receptors can increase the activity of 5-HT3 receptors [47]. This strongly supports the involvement of serotonin receptors in peripheral antinociception induced by BDS 391.

Taken together, the results herein presented demonstrate that the sea anemone *Bunodosoma cangicum* venom’s low molecular weight compound BDS 391, induces, in models of acute and chronic pain, a potent peripheral antinociceptive effect. The pharmacological approaches also indicate that activation of peripheral 5-HT3 receptors contributes to antinociception induced by this compound. Despite the controversial data on the effect of serotonin and serotoninergic receptors, located in CNS or at the periphery, on the nociceptive system, the results herein obtained on the antinociceptive action of BDS 391 as well as of Biguanide support a role for 5-HT3 on pain control. In contrast to the well characterized biological activities of peptides obtained from sea anemone venoms, the roles of low-molecular weight compounds isolated from these venoms are not fully established. Regarding the effect of anemone toxins on the nociceptive system, Andreev et al. (2008) [70] demonstrated that a polypeptide of 56 amino acid residues (APHC1), obtained from the venom of the anemone Heteractis Crispa, induces antinociception by a mechanism dependent on TRPV1 receptors. Our study is the first one to show that a non-peptidic low molecular weight compound obtained from a sea anemone is able to induce antinociceptive activity by activation of peripheral 5-HT3 receptors.

## 4. Material and Methods

### 4.1. Animals

Male Wistar rats (160–180 g) and Swiss mice (18–22 g) from the Instituto Butantan Animal Care Facility were used in this study. Animals were housed in a temperature (21 ± 2 °C) and light (12/12 h light/dark cycle) controlled room. The behavioral tests were carried out between 9:00 am and 4:00 pm. Available *ad libitum* were standard for food and water. All procedures followed the guidelines published by the International Association for Study of Pain regarding the ethical use of conscious animals in pain research (Zimmermann, 1983) [71], all approved by the Institutional Animal Care Committee of the Butantan Institute (CEUAIB, protocol number 494/08, 11/06/2008).

### 4.2. Formalin Test

Twenty microliters of 1% formalin (Shynth, Diadema, SP, Brazil) (prepared in 0.85% saline) were subcutaneously injected into the dorsal hind paw of Swiss male mice. After injection, the animals were immediately placed in a glass cylinder for observation [72]. The cylinders were placed in front of a mirror to allow for full animal observation during the test. The duration of reaction time (paw licking or biting), using a chronometer, between 0–10 min (first phase) and 20–50 min (second phase) after formalin injection was the means used to determine nociception.

### 4.3. Prostaglandin E_2_-Induced Hyperalgesia

Here, we induced hyperalgesia on rats, through subcutaneous injection into one of the hind paws of 0.1 mL of sterile saline (0.85% NaCl solution) containing prostaglandin E_2_ (PGE_2_, 100 ng/paw, Sigma Chem. Co., St. Louis, MO, USA ), as previously described [73]. Pain threshold was measured before and 3 h after PGE_2_ injection.

### 4.4. Chronic Constriction Injury

To induce neuropathic pain, chronic constriction of the rat sciatic nerve was performed following the description by Bennett and Xie (1988) [74,75]. Halothane was used for rat anesthesia. At the level of the middle of the thigh, the common sciatic nerve was exposed by blunt dissection through biceps femoris. About 7 mm from the sciatic nerve trifurcation, the nerve was freed of adhering tissue, and four ligatures (4.0 chromic gut), with about 1 mm spacing, were tied loosely around it. The ligatures were carefully tied, and so the diameter of the nerve was just barely constricted. The incision was stitched in layers. Sham-operated rats were used as control. For this, the animals had the sciatic nerve exposed, but left unaffected. Neuropathic pain was characterized by the presence of hyperalgesia and allodynia, which were evaluated on Days 1 and 14 after surgery.

### 4.5. Evaluation of Mechanical Hyperalgesia

Increase in nociceptive threshold (hyperalgesia) was determined by the rat paw pressure test [76], as described by Gutierrez et al. (2008) [77]. To assess pressure pain thresholds prior to prostaglandin injection or nerve ligation, and again at different periods after treatment or surgery, an Ugo-Basile pressure apparatus was used. Briefly, an increasing force (in g, 16 g/s) was applied to the hind paw. The force needed to induce paw withdrawal was recorded as the nociceptive threshold. To reduce stress, the rats were habituated to the apparatus and to the experimental procedure on the day before the experiment. Testing was blind concerning group designation.

### 4.6. Evaluation of Low Threshold Mechanical Allodynia

To evaluate low-threshold mechanical allodynia, the modified up-down method [41], using the von Frey test [40], was applied prior to nerve ligation and, again, later, at different periods of time. To determine the stimulus intensity threshold stiffness required to elicit a paw withdrawal response, a logarithmic series of 10 calibrated Semmes-Weinstein monofilaments (von Frey hairs, Stoelting, Wood Dale, IL, USA) was applied to the right hind paw. Log stiffness of the hairs ranged from 3.61 (0.407 g) to 5.18 (15.136 g). The 2.041 g hair was first used to assess the basal line. If paw withdrawal occurred, the same hair was applied again 30–60 s later. In the case a response was detected, the 0.407 g (weakest stimulus) monofilament was applied. Whenever responses occurred to the 0.407 g, this filament was assigned as the lower cut-off value. If no response (paw withdrawal) was detected with this filament, the heaviest next monofilament was used (0.692 g). The monofilament that elicited a clear paw withdrawal response was once again applied, 30–60 s later. If paw withdrawal was observed on two consecutive trials with the same stiffness value, no further von Frey hairs were used. If there was no consecutive response to the initial 2.041 g monofilament, application of filaments—in ascending order—continued until two consecutive responses were detected. If no response was observed to the strongest stimulus (15.136 g), this was considered the cut-off value. Behavioral responses were used to determine the 50% paw withdrawal threshold (tactile threshold), according to the procedures described in the literature [41,42,43,44,45]. In this assay, the rats were habituated to the experimental environment once a day, for four days before the experiments, to reduce stress. Regarding group designation, all behavioral assays were blind.

### 4.7. Pharmacological Treatments

BDS 391 was dissolved in sterile saline and administered by intraplantar (i.pl.) route. Since a large amount of the sea anemone compound is required to perform the pharmacological tests, we used in these tests the synthetic BDS 391, produced as previously described [14]. The antinociceptive effect of the compound was evaluated at different times after treatment. Selective 5-HT1, 5-HT2 and 5-HT3 serotonin receptor antagonists were i.pl. administered 15 min before BDS 391 injection to evaluate the involvement of peripheral serotoninergic receptors in the antinociceptive effect of BDS 391. The doses of the antagonists were chosen based on data obtained in preliminary experiments showing that the antagonists, in these doses, inhibit the antinociceptive effect of the selective agonists of the receptors, but did not interfere, per se, with hyperalgesia induced by PGE_2_ (data not shown). Biguanide, a selective agonist of 5-HT3 receptors, was used as positive control to confirm the antinociceptive activity of 5-HT3 receptors. All the antagonists and agonists were purchased from Sigma Chem. Co., St. Louis, MO, USA.

### 4.8. Statistical Analysis

The results are presented as the mean ± SEM. For the behavioral analysis, statistical evaluation of data was carried out using analysis of variance (ANOVA) with post-hoc testing by Tukey contrast analysis. Values of *p* < 0.05 were considered significant.

### 4.9. Radioligand Binding Assay

The ability of BDS 391 to displace the binding of the radioligands [^3^H] 8-OH-DPAT (1.5 nM), [^3^H] Ketanserin (0.5 nM) or [^3^H] GR-65630 (0.69 nM), antagonists of, respectively, 5-HT1, 5-HT2 and 5-HT3 serotoninergic receptors, was determined by Ricerca Biosciences LCC, (Pharmacology Laboratories 158 Li-The Road, Peitou Taipei, Taiwan 112. Taiwan R.O.C.). Briefly, human recombinant CHO-K1 and HEK 293 cells membranes were incubated for 1 h at 25 °C, with 50 mM Tris-HCl, pH 7.4, 1 mM EDTA, 5 mM MgCl buffer containing the radioligands. Non-specific binding was determined using 10 µM Metergoline, 1 µM Mianserin or 10 µM MDL 72222, respectively. For the dissociation assays, BDS 391 was added to the incubation medium in concentrations up to 2 µM. Scintillation counting employing a Beckman LS6000SC (Fullerton, CA, USA) was used to determine radioactivity. For data (IC values) analysis, a nonlinear, least squares regression analysis was applied, using the MathIQä software (ID Business Solutions Ltd., Guildford, UK). Compound’s concentration able to inhibit radioligand binding by 50% of the maximum (IC_50_ value) was determined for each antagonist.
